# Current Induced Spin-Polarization in Chiral Molecules

**DOI:** 10.1021/acs.jpclett.4c01362

**Published:** 2024-06-10

**Authors:** J. Fransson, L. Turin

**Affiliations:** † Department of Physics and Astronomy, Box 516, 751 20, Uppsala University, Uppsala 751 21, Sweden; ∥ Clore Laboratory, University of Buckingham, Buckingham MK18 1EG, U.K.

## Abstract

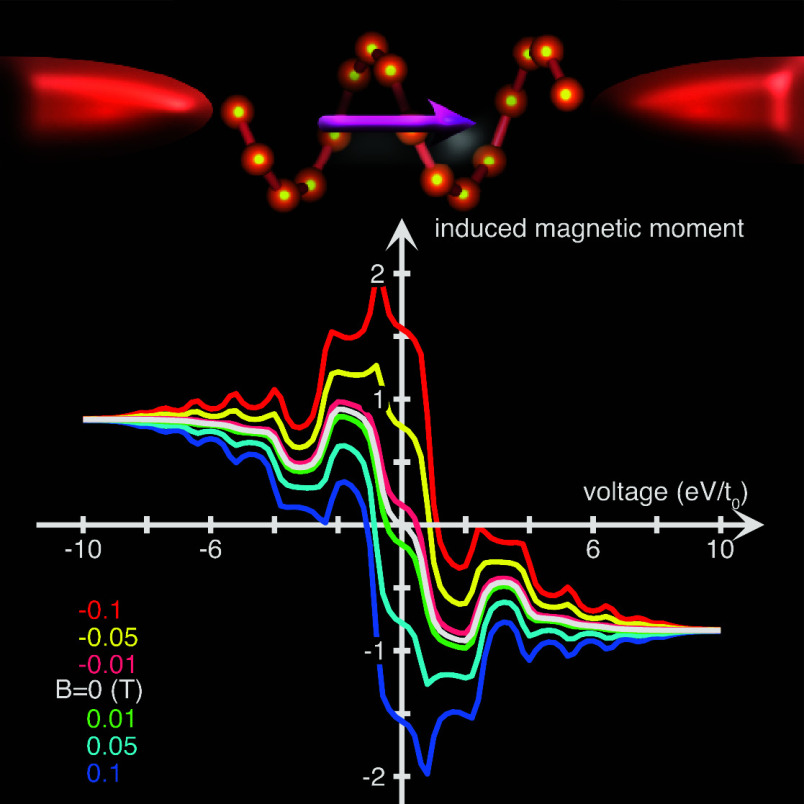

The inverse spin-galvanic effect or current-induced spin-polarization is mainly associated with interfaces between different layers in semiconducting heterostructures, surfaces of metals, and bulk semiconducting materials. Here, we theoretically predict that the inverse spin-galvanic effect should also be present in chiral molecules, as a result of the chiral induced spin selectivity effect. As proof-of-principle, we calculate the nonequilibrium properties of a model system that previously has been successfully used to explain a multitude of aspects related to the chiral induced spin selectivity effect. Here we show that current driven spin-polarization in a chiral molecule gives rise to a magnetic moment that is sensitive to external magnet field. The chiral molecule then behaves like a soft ferromagnet. This, in turn, suggests that magnetic permeability measurement in otherwise nonmagnetic systems may be used noninvasively to detect the presence of spin-polarized currents.

Chirality induced spin selectivity is now a well-established phenomenon in chemistry, physics, and biology.^1^ Whenever electrons flow through a chiral material, it is likely that they will be spin-polarized in the direction of travel.^2^ Aside from its intrinsic interest, this phenomenon has implications both for devices and for our understanding of biological electrochemistry. In aerobes, large electron currents, typically tens of amperes in the resting human, flow through chiral proteins from metabolites to oxygen.^3,4^ What makes spin important in this process of respiration is that the ultimate electron acceptor, dioxygen, is a ground state triplet.

One of the reactions involved in the reduction of oxygen to water involves a two-electron transfer. It has been shown that this reaction is facilitated by spin-polarization.^5^ At a ferromagnetic electrode, oxygen reduction proceeds faster in a magnetic field. Remarkably, general anesthetics, known to affect cellular respiration, markedly reduce spin-polarization at a ferromagnetic electrode.^6^ This said, the extent and importance of spin-polarization in living systems remains unknown. Direct two-terminal measurements of electron current in biology are usually impossible.

Furthermore, almost all measurements thus far have been made under predefined symmetry broken conditions. Transport measurements are done under the conditions that the injected current is spin-polarized, e.g., see refs (7−9). In the case of photoemission, measurements using linearly polarized light were conducted,^10^ demonstrating the spin-polarization of the electrons emitted from a surface of chiral molecules. Recently, the chiral induced spin selectivity effect was put to test in an electron-spin resonance setup in which a chiral molecule was the central component in a donor–acceptor bridge.^11^ This result shows the importance of interfacing to surrounding entities for activating the chiral induced spin selectivity effect, that is, the necessity of a composite structure. There has been little attempt to discern the magnetic properties of chiral structures in the absence of external magnetic boundary conditions. If that were possible, it may amount to a noninvasive method to assess, for example, whether the electron currents flowing in a living organism are spin-polarized.

Here, we relate some recent findings, suggesting that extended chiral molecules can become spin-polarized as a response to the flux of charge through them. This spin-polarization, in turn, varies with the external magnetic field, and this variation is manifested as an anomalous magnetic permeability in the absence of any ferromagnetic material. To show this, we employ a theoretical model^12^ with which a wide range of experimental observations related to the chiral induced spin selectivity effect can be explained.

There is a lively debate on whether the spin angular momentum is being transferred from the substrate into the chiral molecules or whether it is an intrinsic part of the molecule. In fact, the only attempts that have been made to this end relate to the detection of possible Yu–Shiba–Rusinov states,^13^ an electric field induced anomalous Hall effect using chiral molecules,^14,15^ and magneto-resistance measurements perpendicular to the chiral molecules.^9^ Here, we describe some recent findings in this respect, suggesting that chiral molecules should become spin-polarized as a response to the flux of charge through them. To this end, we employ a theoretical model^12^ which accounts for a wide range of experimental observations related to the chiral induced spin selectivity effect, e.g., see refs (8, 13, 16, and 17).

One measurement that has not been done so far is that of the magnetic moment of the chiral molecule in which a spin-degenerate charge current flows. All transport measurements thus far have been performed under conditions where the injected current is spin-polarized, e.g., see refs (7−9), while photoemission measurements using linearly polarized light^10^ have shown that the emitted electrons are spin-polarized. Nevertheless, none of these measurements disclose any unequivocal fact about the nature of the molecules involved.

Current induced spin-polarization has been experimentally observed in different types of heterostructures^18−22^ and doped semiconductors^20^ and on the metallic Pt surface.^23^ Theoretically, current induced spin-polarization has been predicted for two-dimensional electron gas^24−26^ and one-dimensional conduction channels coupled to quantum dots.^27,28^

This article is based on a highly simplified theoretical model,^12^ one of several different proposed to account for the chiral induced spin selectivity effect. While no determination can be made at this point as to which model best reflects the actual physics of the chiral induced spin selectivity effect, our approach is the only one that captures the magnitude of the chiral induced spin selectivity effect, the length dependence,^12,16^ and the temperature dependence,^8,12^ establishing local magnetic moments^13,29^ as well as the angular dependence of the external spin-polarization of the injected electrons.^30^ The results displayed here will also serve as a basis for experiments that may discriminate between the existing theoretical proposals.

We predict that chiral molecules become magnetic or spin-polarized whenever there is a flow of charge current. We also predict that this current induced spin-polarization gives rise to a measurable magnetic susceptibility which may in turn be used to detect the chiral induced spin selectivity effect noninvasively, particularly when no other magnetic species are present, which is frequently the case in biological systems.

Using our model introduced below,^12^ we calculate the charge current (see the inset in Figure 1) through a chiral (helical) molecule comprising 16 sites equidistantly distributed over six laps. The current–voltage characteristics are typical for the current accessing an electronic structure with a limited bandwidth. The electron flux through the chiral molecule induces spin-polarization, which is absent for achiral structures (not shown). For magnetic fields up to 0.1 T applied along the helical axis, the current–voltage shows only negligible variation. By contrast, the current induced molecular spin-polarization varies strongly with the field strength, which is shown in the main panel of Figure 1. Within the range where the current increases, there is a significantly asymmetric response to the magnetic field, in the sense that the induced spin-polarization increases (decreases) for magnetic fields aligned in parallel (antiparallel) with the elongation of the molecule. This property suggests the existence of a current induced magnetic anisotropy, which appears as a hysteresis loop in the voltage dependence of the induced spin-polarization.

**Figure 1:**
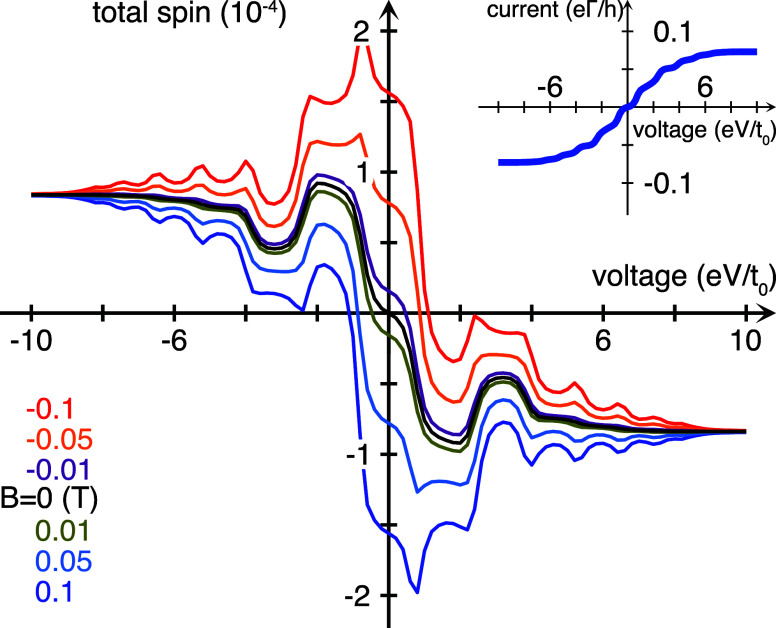
Current induced spin-polarization as a function of the voltage bias for different magnetic fields ranging between ±0.1 T applied to a chiral molecule comprising 2 × 8 sites. The inset shows the corresponding current–voltage characteristics. Here, we have used *t*_0_ = 1 eV, ε_0_–μ = 0, λ_0_ = *t*_0_ × 10^–3^, *t*_1_ = λ_0_/10, 10^–2^ ≤ ω_*n*_/*t*_0_ ≤ 1, *n* = 1, 2, ..., 30, Γ^χ^ = *t*_0_/20, and *T* = 300 K.

As the current saturates, the plateau that emerges for voltages larger than ±10 V, and the induced spin-polarization as well, acquires a constant dependence on the voltage. The existence of the plateau in this setup is a result of the limited number of molecular electron density of states captured within the energy window defined by the difference between the chemical potentials to the left and right of the molecule. Essentially, this means that there is no additional conductance channel available for the charge flux through the molecule.

To be specific, we have used a model captured by the Hamiltonian , where  describes a noninteracting and nonmagnetic electron gas in the left/right (*L*/*R*) lead. Here, ψ_**k**_ = (ψ_**k**↑_ψ_**k**↓_)^*t*^ denotes the electron spinor associated with the energy ε_**k**_. The chiral molecule is modeled as
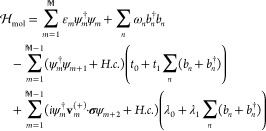
1Here, the first term defines  electron sites with a single electron level ε_*m*_ = ε_0_ – μ = 0 per site, where μ = 0 is the common chemical potential of the junction. These sites are distributed along the helical coordinates **r**_*m*_ = (*a* cos ϕ_*m*_, *a* sin ϕ_*m*_, ¢ϕ_*m*_), where , ¢ = *c*/2π, and *a* and *c* define the radius and length of the helix, respectively. In particular, for a regular quasi-periodic helix , where *M* and *N* denote the number of laps and sites per lap, respectively. The second term in eq 1 includes nuclear vibrations that are created and annihilated by the operators *b*_*m*_^†^ and *b*_*m*_, respectively, at the frequencies corresponding to the energies ω_*m*_. Electrons may transfer between the nearest neighboring and next nearest neighboring sites with elastic rates *t*_0_ and λ_0_, respectively, as well as inelastic rates *t*_1_ and λ_1_. In the last term, the chirality of the molecule is included in the curvature vector **v**_*m*_^(s)^, where *s* = ±. The curvature is coupled to the spin via the Pauli matrices **σ**, hence accounting for an effective spin–orbit coupling in the molecule. The molecules are, finally, coupled to the leads via , where *t*_χ_**k**, χ = *L*, *R*, is a 2 × 2-matrix in order to allow for spin-dependent interfacing.

The effects of the interfaces are parametrized in terms of Γ^χ^ = 2πIm∑_**k**∈χ_|*t*_χ_**_k_**|^2^*g*_χ**k**_^*r*^(ω) defining the coupling between the molecule and the left/right lead, where *g*_χ**k**_^*r*^(ω) is the Green function for an electron in the lead.

The method for calculating physical quantities, such as the density of electron states and spin-densities, as well as charge and spin distributions is using nonequilibrium Green functions and has been addressed in, e.g., refs (12, 29, 31, and 32). Specifically, the charge current *J* is mathematically expressed as
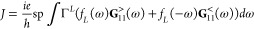
2where **G**_*mn*_^</>^(ω) denotes the lesser/greater electron Green function connecting the sites *m* and *n*. Here, sp denotes the trace over spin 1/2 space. An example of the calculated current is given in the inset in Figure 1. Similarly, the current induced spin-polarization ***S***_Mol_ represents the net of the individual moments as ***S***_Mol_ = ∑_*m*_⟨**S**_*m*_⟩, where the local nonequilibrium magnetic moment
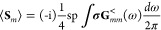
3is physically a measure of the local occupied electron density projected on the Pauli matrices **σ**.

It may be noticed that the nonequilibrium density of occupied and unoccupied states per site *m* are provided by spIm**G**_*mm*_^<^(ω)/2 and −spIm**G**_*mm*_^>^(ω)/2, respectively. In this way, the total density of states DOS_*m*_(ω) = −spIm[**G**_*mm*_^>^(ω) – **G**_*mm*_^<^(ω)]/2, and the spin-projected density **ρ**_*m*_(ω) = −sp**σ**Im[**G**_*mm*_^>^(ω) – **G**_*mm*_^<^(ω)]/4.

The total nonequilibrium density of electron states DOS(ω) = ∑_*m*_DOS_*m*_(ω) and corresponding spin-polarization ρ^*z*^(ω) = ∑_*m*_ρ_*m*_^*z*^(ω) in the molecule corresponding to the current induced magnetic moment plotted in Figure 1 are shown in Figure 2. The narrow low current regime around zero voltage bias, inset of Figure 1, corresponds to a finite density of electron states near the common chemical potential, μ = 0, of the system; see Figure 2(a).

**Figure 2:**
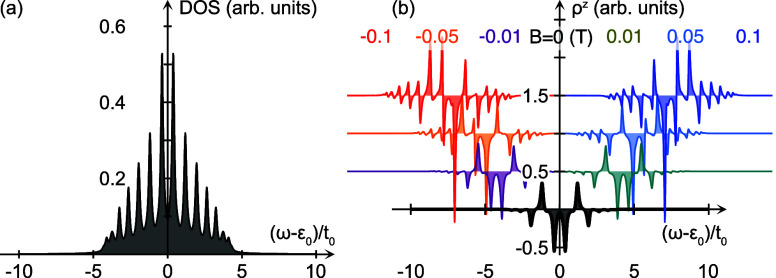
(a) Total nonequilibrium density of electron states DOS(ω) = ∑_*m*_DOS_*m*_(ω) corresponding to the 2 × 8 sites helix in Figure 1 at *B* = 0 and a source-drain voltage of 1 V. (b) Corresponding total nonequilibrium spin-polarization ρ^*z*^(ω) = ∑_*m*_ρ_*m*_^*z*^(ω) for external magnetic fields ranging between ±0.1 T. In panel (b), the plots are offset for clarity.

The spin-polarization ρ^*z*^ is nonvanishing in the absence of the external magnetic field, Figure 2(b) (black), which is a prerequisite for the current induced magnetic moments to emerge. The energy variations of the spin-polarization, repeatedly switching sign, explain the nonmonotonic increase of the total magnetic moment with increasing voltage. The external magnetic field introduces Zeeman splittings in the spectrum which break the symmetry of the spin-polarization around ω = ε_0_, such that a positive (negative) field leads to a slight spin accumulation below (above) this point. In turn, this leads to the total magnetic moment switching sign at negative (positive) voltages; see Figure 1. At sufficiently large voltage bias, the induced total magnetic moment is unaffected by the external magnetic field, see Figure 1, which follows from the fact that the two conditions are simultaneously met. First, for a sufficiently large voltage bias, the total density of electron states is captured in the window between the chemical potentials opened by the voltage, such that there is no additional conduction channel that can open with increasing voltage. Second, the spin-polarization induced by the external magnetic field is negligible compared to the spin-polarization induced by the current. The effect of the external magnetic field is, thereby, reduced to redistribute the spin-densities relative to each other, without generating any additional imbalance between the spin-channels. Hence, simultaneously as the total electron density becomes captured within the voltage window, so does the total spin-polarization. The total imbalance between the spin-channels is, therefore, restored, which leads to the total magnetic moment approaching the same value as if there were no magnetic field applied.

The plots in Figure 3 show a summary of the current induced spin properties in a helical 2 × 8-sites molecule wound clockwise (cw – red) and counterclockwise (ccw – blue) at a voltage bias of 1 V. The amplitude of the total magnetic moment per site |⟨**S**_*m*_⟩| is plotted in Figure 3(a), with the corresponding charge distribution shown in the inset of Figure 3(c). The electric polarization induced by the bias conditions is clearly shown in the charge distribution, which is accompanied by an induced spin-polarization in accordance with the theoretical discussion in refs (29 and 32). The projected spins per site along the *z*-, *x*-, and *y*-directions are shown in Figure 3 (b–d), respectively. Despite the bias, the *z*-projected spin nearly forms a singlet-like configuration Figure 3(b), however, with a slight imbalance tending toward an overweight in the down (up) direction at the right end of the cw (ccw) helix compared to the up (down) spin in the left. Although the spin projections in the *x*- and *y*-directions are nearly 2 orders of magnitude smaller compared to the projection in the *z*-direction, the plots in Figure 3(c and d) also display a similar type of imbalance toward the right of the molecule.

**Figure 3:**
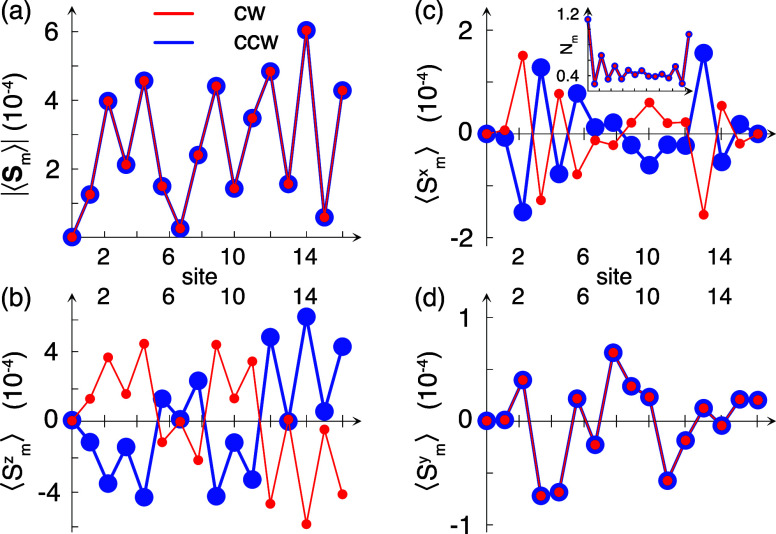
Site resolved current induced spin-polarization for clockwise (cw – red) and counterclockwise (ccw – blue) helices with 2 × 8 sites at the bias voltage *V* = 1 V. (a) Total moment |⟨**S**_*m*_⟩|, (b–d) ⟨*S*_*m*_^*z*^⟩, ⟨*S*_*m*_^*x*^⟩, and ⟨*S*_*m*_^*y*^⟩. Inset in panel (c) shows the corresponding charge densities *N*_*m*_. Other parameters are given in Figure 1.

It should also be noticed that the two enantiomers acquire opposite spin polarizations such that the spin moments ⟨**S**_*m*_⟩_±_ = (⟨*S*_*m*_^*x*^⟩_±_,⟨*S*_*m*_^*y*^⟩_±_,⟨*S*_*m*_^*z*^⟩_±_) in the clockwise (*+*) and counterclockwise (*−*) enantiomers, respectively, are related by rotating the spins around the *y*-projection. Hence, (⟨*S*_*m*_^*x*^⟩_–_,⟨*S*_*m*_^*y*^⟩_–_,⟨*S*_*m*_^*z*^⟩_–_) = (−⟨*S*_*m*_^*x*^⟩_+_,⟨*S*_*m*_^*y*^⟩_+_,–⟨*S*_*m*_^*z*^⟩_+_) .

The current induced spin-polarization tends to increase with increasing length of the chiral molecule, which is illustrated in Figure 4(a) – red. As is also shown, the longitudinal spin susceptibility, *dS*_Mol_/*dB*_*z*_ – purple, accompanies this increase. At the same time, the transverse spin susceptibility, *dS*_Mol_/*dB*_*x*_, is more or less constant as the molecular length increases; see inset of Figure 4(a). Moreover, the spin susceptibility depends on the angle θ between the length direction of the molecule and the direction of the externally applied magnetic field; see the inset of Figure 4(b). This variation is shown in Figure 4(b) for a 4 × 8-sites molecule, which has a striking resemblance with the variation given by cos^2^(θ/2).

**Figure 4:**
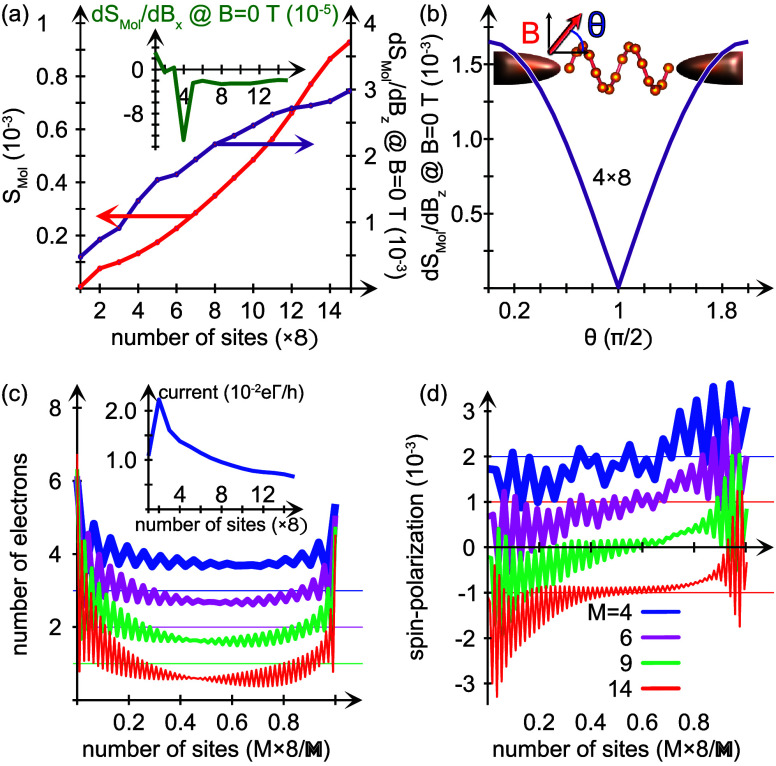
Nonequilibrium properties as a function of molecular length  and a voltage bias of 1 V. (a) Induced magnetic moment *S*_Mol_ (red) and corresponding longitudinal spin susceptibility *dS*_Mol_/*dB*_*z*_ (purple) and (b) spin-susceptibility as a function of polar angle (θ) orientation of the magnetic field relative to the length direction of the molecule, of length 4 × 8. (c) Site resolved charge distributions for *M* = 4, 6, 9, and 14, and (d) corresponding site-resolved spin-polarization. The insets in panels (a), (b), and (c) show the transverse spin susceptibility *dS*_Mol_/*dB*_*x*_, a schematic of the setup, and the charge current, respectively. The plots in panels (c) and (d) are offset for clarity. Other parameters are as shown in Figure 1.

The increased current induced magnetic moment with increasing molecular length can be explained by the internal charge and spin-polarization of the molecule increasing with length; see Figure 4(c and d), respectively. For the shorter chain (blue), the charge polarization is already pronounced and, therefore, the molecule acquires an internal spin-polarization, since it results from the internal charge redistribution that follows upon perturbing the systems.^29,32^ However, for the molecule to acquire a finite magnetic moment, the internal spin-polarization has to be unevenly distributed with respect to the positive and negative contributions, and the greater the discrepancy between these contributions becomes, the larger the resulting magnetic moment. It can be seen from the plots of both charge and spin-polarizations, Figure 4(c and d), that the molecule polarizes in proportion to its length and that the imbalance between the positive and negative spin contributions is enhanced. It is clear, therefore, that the resulting magnetic moment increases with increasing length. The amplitude of the charge current can also be seen to decrease with increasing length, as expected;^33^ see inset of Figure 4(c). This too is related to the electric polarization of the molecular electronic structure, see Figure 4(c), causing the density to become decreasingly conjugated with length.

Before ending this Letter, we make three remarks. First, while we, in this study, consider the typical transport setup with a molecule mounted between two metals, it is, nonetheless, relevant to ask whether our results apply to photoemission spectroscopy,^10^ since for this and similar experiments there is no magnetized substrate involved. By extrapolating the result in the current Letter along with the results in ref (29), it is reasonable that the presence of a metallic electrode leads to breaking the spin symmetry of the molecule. An implication is, hence, that the photoemitted electrons traveling through a chiral molecule pick up this symmetry breaking and, therefore, spin-polarize.

Second, the inclusion of the magnetic field in our present study leads to a Zeeman splitting of the molecular energy levels by at most 0.01 meV, which corresponds to a temperature of about 0.1 K. Such a small spin-polarization cannot be discerned in a room temperature experiment, which our calculations represent. Moreover, the application of an external magnetic field is different from setting up the experiment with a magnetized electrode from which a spin-polarized current emerges; see, for instance, refs (12 and 31).

Third, in the setup with a magnetized electrode, the magnetization is switched using an external magnetic field, which leads to very distinct configurations. The comparison of the charge currents in different configurations, e.g., magnetization up and down, provides a measure of the anisotropic response to the altered conditions, unfortunately referred to as the spin-polarization. This measure is typically presented as the ratio between the difference and the sum of the currents measured in the two configurations. In this Letter, the molecule develops a true spin-polarization as a response to the charge current, meaning that there is an imbalance between carriers with different spins.

In this Letter we predict that a chiral molecule acquires a magnetic moment when a charge current is driven through it, Figure 1. The electric field, or voltage bias, applied across the molecule leads to a charge redistribution and an electric polarization, Figures 3 and 4. Because of the chiral induced spin selectivity effect, the charge redistribution is accompanied by a spin-redistribution that, in turn, generates a net internal spin-polarization of the molecule. Quantitatively, the spin-polarization acquires opposite signs at the two ends of the molecule, hence breaking up the equilibrium molecular spin-singlet state. In particular, the molecular charge polarization can be understood as an imbalance of the charge accumulations at the two ends of the molecule near the interfaces to the leads. This imbalance leads to a corresponding imbalance in the spin accumulations such that its positive and negative contributions do not cancel each other, which results in a nonvanishing molecular magnetic moment.

To the best of our knowledge, our theoretical prediction of the current induced spin-polarization for chiral molecules is novel. This prediction is important since it opens a novel scope in the context of the chiral induced spin selectivity effect as well as new directions for spintronics, electrochemistry, and noninvasive measurements in biological tissues. Importantly, the averaged spin-susceptibility should be nonvanishing even for an isotropically distributed set of molecules, as suggested in Figure 4(b). Therefore, our prediction should be viable also for measurements of magnetic responses of biological tissue where currents are likely to be isotropically distributed.

## Supporting Information Available

Transparent Peer Review report available (PDF)
