# Supplementary material for: Current Induced Spin-Polarization in Chiral Molecules
Source: J Phys Chem Lett. 2024 Jun 10;15(24):6370–4. doi: 10.1021/acs.jpclett.4c01362 (PMC11194818; doi:10.1021/acs.jpclett.4c01362)
Supplement: Supplementary file 1 — jz4c01362_si_001.pdf [file jz4c01362_si_001.pdf]

jz-2024-01362v.R1

Name: Peer Review Information for "Current Induced Spin-Polarization in Chiral Molecules"

First Round of Reviewer Comments

Reviewer: 1

Comments to the Author

The paper utilizes a theoretical model to demonstrate that a magnetic moment is generated within chiral molecules when a charge current flows through it, even when no other magnetic species are present. The spin-polarization is sensitive on the external magnetic field, and is attributed by an imbalance of the charge accumulations at the two ends of the molecule. These results can be used to explain previous experiments related to the CISS effect and become a basis for new experiments to further understand its mechanism and implicancies.

The paper is clear and of particular interest to the wider community working with the CISS effect. I recommend its publication subject to the following revisions:

1) The text refers to the upper and lower inset of Fig 1, but the Figure contains only one inset, corresponding to the current vs voltage curve. Please revise the Figure to include all the information.

Page 5: "By contrast, the current induced molecular spin-polarization varies strongly with the field strength, which is shown in the main panel and the *upper* inset of Fig. 1."

Page 7: "An example of the calculated current is given in the *lower* inset in Fig. 1."

2) From Figure 3, it is not clear why the y-projected spin is the same in both cw and ccw helices.

3) Please check the manuscript for some typos and other small editing mistakes. For example:

i) Page 1: "In aerobes large electron currents, [...] flow through chiral proteins from *metabolism* to oxygen." Do you mean metabolites?

ii) Page 1: "Remarkably, general anesthetics, know to affect cellular respiration..." should be "knowN to affect".

iii) Page 6: "These sites are istributed", a D is missing.

Reviewer: 2

Comments to the Author

The paper by Fransson and Turin theore2cally inves2gates the induced spin polariza2on of current through chiral molecules placed on an interface when driven by charge currents. The authors link their findings to the chiral induced spin selec2vity (CISS) effect, a rela2vely new phenomenon that links electron spin with molecular chirality. The paper is well wriEen, and the theore2cal analysis is sound. The authors' analysis contributes to the theore2cal understanding of the CISS effect, addressing a previously missing link in the field. While the paper does not offer new theore2cal insights and follows the concepts in Fransson's earlier works, it examines an unexplored property of chiral molecules using exis2ng theore2cal models in a simple manner, providing insights for new experiments. **Therefore, I recommend the paper for publica5on, but I kindly request the authors to address the following ques5ons and concerns in the manuscript where necessary:**

- Photoemission measurements (Göhler et al., *Science*, 2011, and others) indicate minimal to no difference in spin polariza2on when conducted using either linearly or circularly polarized light. Addi2onally, electron currents emiEed by linearly polarized light (comprising ½ leR circularly polarized (LCP) and ½ right circularly polarized (RCP) components) should contain equal amounts of leR and right longitudinally polarized photoelectrons. Could the authors clarify how chiral symmetry is broken in this scenario? Specifically, how is spin degeneracy broken?

- The analysis examines an interface involving chiral molecules on a surface. But is this necessary for understanding the CISS effect? Recent experiments (Eckvahl et al., *Science*, 2023) suggest that the chiral molecular structure alone (without an interface or surface) is sufficient to achieve spin polariza2on. Given this, wouldn't theore2cal models be simpler if free chiral molecules (without leads) were exposed to an external electric field?

- From my understanding, the primary finding of the paper is the anisotropic S/V characteris2c when an external field is applied (as in Figure 1). Does this imply that the authors have merely incorporated an external field into their previous calcula2ons (as seen in Refs 27, 29, and 30)? For the sake of clarity and to highlight the significance of the manuscript, it would be beneficial if the authors explicitly explained how their current analysis diverges from their previous work.

- The spin polariza2on values calculated by the authors (approximately 0.01% as shown in Figure 1) remain significantly different from the observed values. More cri2cally, these values

are even lower than those reported in Fransson's earlier work (Ref 29), which u2lized the same theore2cal model. How can this discrepancy be explained?

In addi2on to these ques2ons, I suggest the authors consider ci2ng the following papers:

1. The first direct observa2on of CISS without an interface by the Wasielewski group (Eckvahl et al., 2023).
2. Recent work by Ozturk and Sasselov (Ozturk et al., 2023) combining the origin of life's chemistry and CISS, which also relies on currentinduced spin polariza2on near surfaces, has important implica2ons for biology's single-handedness. Can go to the introduc2on.
3. Recent work by Niman et al. (2023) on the spin selec2vity of bacterial interfaces in extracellular electron transfer. The model the authors present may be relevant to this type of biological electron transfer. Can go to the introduc2on.

Author's Response to Peer Review Comments:

Institutionen för Fysik och Astronomi
Dr. Jonas Fransson
Professor

Besöksadress:
Ångströmlaboratoriet,
Polacksbacken
Lägerhyddvägen 5

Postadress:
Box 516
751 21 Uppsala

Telefon:
018 471 5864

Telefax:
018 471 3524

Mobil:
070 167 9264

Hemsida:
http://www.physics.uu.se

Epost:
Jonas.Fransson@physics.uu.se

Dear Editor

We hereby resubmit our manuscript *Chiral Phonon Induced Spin-Polarization*, by J. Fransson and L. Turin, for consideration for publication in the Journal of Physical Chemistry Letters.

We are very pleased to see the positive reactions from the reviewers. While they both have asked a few questions, none of them need to review the manuscript again. Below, however, we have responded to each reviewer in detail and we also indicate the minor revision we have done and where.

Therefore, we hope you will agree that our paper is suitable for publication in the Journal of Physical Chemistry Letters.

Yours sincerely,

Jonas Fransson

Department of Physics and Astronomy
Jonas Fransson
Professor

Visiting address:
Ångströmlaboratoriet,
Polacksbacken
Lägerhyddvägen 5

Postal address:
Box 516
SE-751 21 Uppsala
SWEDEN

Telephone:
+46 18 471 5864

Telefax:
+46 18 471 3524

Cell:
+46 70 167 9264

Web page:
http://www.physics.uu.se

Email:
Jonas.Fransson@physics.uu.se

Response to the first reviewer

We thank the reviewer for the kind words about our manuscript and that they see the potential for new experiments. We also respond in detail to the comments raised.

1. Reviewer comment:

   The text refers to the upper and lower inset of Fig 1, but the Figure contains only one inset, corresponding to the current vs voltage curve. Please revise the Figure to include all the information.
   *Page 5: "By contrast, the current induced molecular spin-polarization varies strongly with the field strength, which is shown in the main panel and the *upper* inset of Fig. 1."*
   *Page 7: "An example of the calculated current is given in the *lower* inset in Fig. 1."*

   We thank the reviewer for the careful reading, pointing the typos and mistakes in the text. These flaws have been corrected.

2. Reviewer comment:

   From Figure 3, it is not clear why the y-projected spin is the same in both cw and ccw helices.

   The reviewer correctly points out that the y-projected spin is the same in both enantiomers. The same behavior of the spin projections was reported also in *J. Phys. Chem. Lett.* **2022**, *13*, 808-814, and has to do with set-up of the chiral structure. The two helices/enantiomers are defined by rotations clockwise of counter-clockwise around the z-axis. This is equivalent to rotating the spin coordinate system around its y-axis such that the spin vector (S_x, S_y, S_z) is rotated into $(-S_x, S_y, -S_z)$. Hence, the sign of the y-projection remains unchanged.

   We have added a brief explanation for this around Fig. 3.

3. Reviewer comment:

   Please check the manuscript for some typos and other small editing mistakes. For example:
   *i) Page 1: "In aerobes large electron currents, [...] flow through chiral proteins from *metabolism* to oxygen." Do you mean metabolites?*
   ii) Page 1: "Remarkably, general anesthetics, know to affect cellular respiration...should be "knowN to affect".
   iii) Page 6: "These sites are istributed", a D is missing

   The reviewer points out a few mistakes which we have corrected. We thank the reviewer for the careful reading.

Response to the second reviewer

We thank the reviewer for the kind words about our manuscript, that this work contributes to the theoretical understanding of the chiral induced spin selectivity effect. We also respond in detail to the comments raised.

1. Reviewer comment:

   Photoemission measurements (Göhler et al., Science, 2011, and others) indicate minimal to no difference in spin polarization when conducted using either linearly or circularly polarized light. Additionally, electron currents emitted by linearly polarized light (comprising 1/2 left circularly polarized (LCP) and 1/2 right circularly polarized (RCP) components) should contain equal amounts of left and right longitudinally polarized photoelectrons. Could the authors clarify how chiral symmetry is broken in this scenario? Specifically, how is spin degeneracy broken?

   It is surely a very good question and we believe that somehow the answer lies within the framework of our results. However, the scope of the manuscript is not to explain any specific observation. Nevertheless, the fact that the photoemission results point towards a minor difference between illuminating with linearly and circularly polarized light may be connected to our finding. We thank the reviewer for raising this issues and we have added some comments about this in the discussions section.

2. Reviewer comment:

   The analysis examines an interface involving chiral molecules on a surface. But is this necessary for understanding the CISS effect? Recent experiments (Eckvahl et al., Science, 2023) suggest that the chiral molecular structure alone (without an interface or surface) is sufficient to achieve spin polarization. Given this, wouldn't theoretical models be simpler if free chiral molecules (without leads) were exposed to an external electric field?

   Here, it is important to be clear about the differences between isolated chiral molecules and chiral molecules connected to a surrounding. The experiment by Eckvahl et al., is by no means a demonstration that there is a chiral induced spin selectivity effect in the chiral molecule alone. In their experiment the chiral molecule is playing the central part of a donor-acceptor bridge without which the experiment cannot be performed. It is not the presence of a metal that is necessary, it is breaking the closed shell structure of the chiral molecule which is central. In other words, it is the compositeness that is essential, not the specific nature of the connecting part. It is, nevertheless, possible to construct an effective model for the chiral molecule in which the connections to a surrounding is included, and it is important to realize that the chiral induced spin selectivity effect does not arise without such connection to another entity.

   We have added the reference per the reviewer's suggestion and pointed out the necessity of connecting the chiral molecule to something.

3. Reviewer comment:

   From my understanding, the primary finding of the paper is the anisotropic S/V characteristic when an external field is applied (as in Figure 1). Does this imply that the authors have merely incorporated an external field into their previous calculations (as seen in Refs 27, 29, and 30)? For the sake of clarity and to highlight the significance of the manuscript, it would be beneficial if the authors explicitly explained how their current analysis diverges from their previous work.

   We thank the reviewer for raising this issue since there is a quite important difference between the set-ups in the present manuscript and the previous papers. The magnetic field introduces a Zeeman splitting of the molecular levels which is of the order of about 1 meV, or about 1 K. This is a far too small splitting to have any bearing on the chiral induced spin selectivity effect. In the conventional transport set-up, there is a spin-polarized electron density in either the source or the drain which acts through exchange with the electrons in the molecule. The anisotropic response to this spin-polarization can be rather large and, clearly, measurable at room temperature.

   We have added a few sentences about these differences in the discussions section at the end of the manuscript which we hope clarifies this issue.

4. Reviewer comment:

   The spin polarization values calculated by the authors (approximately 0.01 % as shown in Figure 1) remain significantly different from the observed values. More critically, these values are even lower than those reported in Fransson's earlier work (Ref 29), which utilized the same theoretical model. How can this discrepancy be explained?

   This question is related to the previous and demonstrates very well that we have to be clearer with the set-up of the model. In the current manuscript, a true spin-polarization develops as a response to the charge current flowing through the molecule. And yes, this spin-polarization is small measured in units of the Bohr magneton. There is no relative measure in this set-up, which is the typical situation in the transport measurements of the chiral induced spin selectivity effect. Here, there is no spin-polarized current injected from the reservoirs and we do not compare two current with each other.

   This difference in the set-up and expectation have been addressed in the discussions section at the end of the manuscript.

5. The reviewer also asks us to cite three more papers, of which we have added two, Eckvahl et al., 2023, and Niman et al., 2023. We have, however, judged that the references Ozturk, et al., 2023, is not relevant in our context.

Additions to the text

1. Third paragraph on page 3:
   Recently, the chiral induced spin selectivity effect was put to test in an electron-spin resonance set-up, in which a chiral molecule was as the central component in a donor-acceptor bridge.[11] This result shows the importance of interfacing to surrounding entities for activating the chiral induced spin selectivity effect, that is, the necessity of a composite structure.

2. Page 10:
   Before ending this Letter, we make a three remarks. First, while we in this study consider the typical transport set-up with a molecule mounted between two metals, it is, nonetheless, relevant to ask whether our results apply to the photo-emission spectroscopy[10] since this and similar experiments there is no magnetized substrate involved. By extrapolating the result in the current Letter along with the results in Ref. [29], it is reasonable that the presence of a metallic electrode leads to breaking the spin symmetry of the molecule. An implication is, hence, that the photoemitted electrons traveling through a chiral molecule pick up this symmetry breaking and, therefore, spin-polarize.
   Second, the inclusion of the magnetic field in our present study leads to a Zeeman splitting of the molecular energy levels by at most 0.01 meV, which corresponds to a temperature of about 0.1 K. Such a small spin-polarization cannot be discerned in a room temperature experiment, which our calculations represent. Moreover, the application of an external magnetic field is different from setting up the experiment with a magnetized electrode from which a spin-polarized current emerges, see, for instance, Refs. [12,31].
   Third, in the set-up with a magnetized electrode, the magnetization is switched using an external magnetic field which leads to very distinct configurations. The comparison of the charge currents in different configurations, e.g., magnetization up and down, provides a measure of the anisotropic response to the altered conditions, unfortunately referred to as the spin-polarization. This measure is typically presented as the ratio between the difference and the sum of the currents measured in the two configurations. In this Letter, the molecule develops a true spin-polarization as a response to the charge current, meaning that there is an imbalance between carriers with different spins.
